# Supplementary material for: Clinical Course, Outcomes, and Risk Factors of Myocarditis and Pericarditis Following Administration of mRNA-1273 Vaccination: A Protocol for a Federated Real-World Evidence Vaccine Safety Study Using Data from Five European Data Sources
Source: Vaccines (Basel). 2025 Jul 16;13(7):755. doi: 10.3390/vaccines13070755 (PMC12299067; doi:10.3390/vaccines13070755)
Supplement: Supplementary file 1 [file vaccines-13-00755-s001.zip › vaccines-3702835-supplementary.pdf]

SUPPLEMENTARY INFORMATION

Clinical course, outcomes and risk factors of myocarditis and pericarditis following administration of mRNA-1273 vaccination: a protocol for a federated real-world evidence vaccine safety study using data from five European data sources

Laura C. Zwiers[1,2,*], Diederick E. Grobbee[1,2], Rob Schneijdenberg[2], Corine Baljé[3], Samantha St Laurent[4], Daina B. Esposito[4], Lei Zhu[4], Veronica V. Urdaneta[4], Magalie Emilebacker[4], Daniel Weibel[5], Felipe Villalobos[6], Carlo Alberto Bissacco[6], Arantxa Urchueguía Fornes[7,8], Juan José Carreras Martínez[7,8], Anteneh Assefa Desalegn[9], Angela Lupattelli[9], Lei Wang[10], Jannik Wheler[10], Vera Ehrenstein[10], Denise Morris[11,12], Catherine Fry[11,12], Marjolein Jansen[2], Brianna M. Goodale[2], Julius Global Health, Julius Center for Health Sciences and Primary Care, University Medical Center Utrecht, Utrecht University, Utrecht, The Netherlands;

1 Julius Clinical, Zeist, The Netherlands;
2 Clin-Q B.V., Groningen, The Netherlands;
3 ModernaTX, Inc., Cambridge, Massachusetts, USA;
4 Vaccine Monitoring Collaboration for Europe (VAC4EU), Brussels, Belgium;
5 Fundació Institut Universitari per a la recerca a l'Atenció Primària de Salut Jordi Gol i Gurina (IDIAPJGol), Barcelona, Spain;
6 Vaccine Research Department, Foundation for the Promotion of Health and Biomedical Research in the Valencian Region (FISABIO - Public Health);
7 CIBER de Epidemiología y Salud Pública, Instituto de Salud Carlos III;
8 Pharmacoepidemiology and Drug Safety Research Group, Department of Pharmacy, Faculty of Mathematics and Natural Sciences, University of Oslo, Oslo, Norway;
9 Department of Clinical Epidemiology, Aarhus University and Aarhus University Hospital, Aarhus, Denmark;
10 Drug Safety Research Unit, Southampton, UK;
11 University of Portsmouth, Portsmouth, UK;
12 Department of Medical Microbiology and Infection Control, Franciscus Gasthuis & Vlietland, Rotterdam, The Netherlands;
* Correspondence: l.c.zwiers-3@umcutrecht.nl

Supplementary Table S1. Overview of all determinants of interest and how they will be operationalised in the study*

Variable	Operationalisation
Sex	Sex (male, female, other) recorded at index date
Age	Age measured in years at index date
Vaccination characteristics	
Number of doses	Total number of mRNA-1273 doses received
Most recent vaccination prior to mRNA-1273	The most recent vaccination received prior to the mRNA-1273 vaccine.
Inter-dosing interval	Number of days between two doses of the mRNA-1273 vaccine
Healthcare utilisation and medical history	
Inpatient hospital visit	Indicator of whether an individual had an inpatient hospital visit in the year prior to index date
Inpatient visit duration	Total number of days spent in hospital in the year prior to index date
ICU admissions	Indicator of whether an individual was admitted to the ICU in the year prior to index date
Outpatient hospital visits	Indicator of whether an individual had an outpatient hospital visit in the year prior to index date
Emergency room visits	Indicator of whether an individual had an emergency room visit in the year prior to index date; not available in Norwegian registry data
Primary care visits	Number of GP visits in the year prior to index date
Comorbidities	For all comorbidities listed in Table A2, we will assess whether there is a record of them in the two years prior to index date
Charlson Comorbidity Index	Charlson Comorbidity Index [33], calculated at index date
Medical treatments	For all medical treatments listed in Table A3, we will assess whether there is a record of them in the two years prior to index date
Previous SARS-CoV-2	Indicator of whether an individual tested positive for SARS-CoV-2 in the two years prior to index date
Time since last SARS-CoV-2 episode	Number of days between most recent SARS-CoV-2 positive test and index date
Non-COVID vaccines	For all vaccines listed in Table A4, we will assess whether these were administered in the year prior to index date
Lifestyle variables (all included based on availability within DEAPs)	
BMI	Numerical BMI measured during the year prior to index date; only available in data from CPRD Aurum and SIDIAP
Weight status	Weight status (underweight, normal, overweight, obese), recorded during year prior to index date; only available in data from SIDIAP
Smoking	Self-reported smoking status (never, former, current), recorded during year prior to index date; only available in data from CPRD Aurum and SIDIAP
Alcohol consumption	Self-reported number of units of alcohol consumed weekly, recorded during year prior to index date; only available in data from SIDIAP
Alcohol abuse	Indicator of whether an individual was reported to suffer from alcohol abuse; only available in data from CPRD Aurum
Physical activity	Self-reported weekly hours of physical activity, recorded during year prior to index date; only available in data from SIDIAP
Employment status	Employment status during year prior to index date; only available in Norwegian registry data
Yearly gross income	Yearly gross income during year prior to index date; only available in Norwegian registry data
Socioeconomic Status indicator: MEDEA deprivation index	Level of the MEDEA deprivation index [39] in year prior to index date; only available in data from SIDIAP

*Unless otherwise specified in the table, the variable will be operationalised similarly across all DEAPs
ICU = Intensive Care Unit; BMI = Body Mass Index

Supplementary Table S2. List of all comorbidities of interest

Anaemia
Coagulation deficiencies
Sickle Cell Disease
Cardiocerebrovascular disease
Cardiomyopathy
Cardiovascular disease
Heart failure including chronic HF
Valvular heart disease
Angina
Arrhythmia
Endocarditis
Coronary artery disease
Myocarditis (registered >6months before vaccination)
Pericarditis (registered >6months before vaccination)
Alcoholic chronic liver disease
Hepatitis autoimmune
Inflammatory bowel disease
Liver chronic disease
Liver cirrhosis
Non-alcoholic fatty liver disease
Peptic ulcer disease
Diabetes (types 1 and 2)
Gout
Kidney disease, chronic
Thyroiditis, autoimmune
AIDS
HIV
Influenza
Infections, respiratory tract
Anaphylaxis history
Autoimmune disorders
Immunodeficiencies
Sjögren's syndrome
Systemic lupus erythematosus
Organ transplant recipient
Connective tissue disease
Psoriatic arthritis
Arthritis, rheumatoid
Polymyalgia rheumatica
Alcohol abuse
Sleep disturbance, insomnia
Substance use disorders
Haemorrhage, cerebrovascular
Dementia all
Hemiplegia
Weight loss, anorexia
Cachexia
Any malignant cancer or tumour
Chronic respiratory disease
Psoriasis

Hypertension
Vascular disease, peripheral
Renovascular disease
Vasculitis
Scleroderma
Asthma
COPD
Respiratory insufficiency

Supplementary Table S3. List of medical treatments of interest

Angiotensin converting enzyme inhibitors
Agents acting on the renin-angiotensin system
Antiarrhythmics
Antibiotics
Anticoagulants (warfarin and novel oral anticoagulants)
Antithrombotic agents
Antiviral medications
Angiotensin receptor blockers
Aspirin low dose
Corticosteroids
Beta blockers
Bronchodilators
Cancer hormones
Cardiac Stimulants
Calcium channel blockers
Antihypertensives
Antineoplasic agents
Cardio/cerebrovascular disease drugs
Cardiovascular disease drugs
Erythropoietin
Diabetes drugs
Antiviral agents for the treatment of HCV infections
Drugs used for chronic respiratory diseases
Drugs used for sickle cell disease
Digoxin
Diuretics
Heparins
Immunostimulants
Immunosuppressants
Insulin
Drugs used in nicotine dependence
Nitrates
Novel oral anticoagulants and Platelet aggregation inhibitors excluding heparins
Nonsteroidal anti-inflammatory drugs
Oral glucose lowering drugs
Other antihypertensive
Other cardiac preparations
Other respiratory drugs
Platelet aggregation inhibitors
Psychotropics Psycholeptics
Statins
Warfarin
Clozapine
Epinephrine

Supplementary Table S4. List of vaccines not targeting SARS-CoV-2 of interest, with indicators for whether they are included in the data of different DEAPs

	**Danish registries**[1]	**Norwegian registries**[2]	**VID**	**SIDIAP**	**CPRD Aurum**[3]
Influenza containing vaccines	Included	Included	Included	Included	Included
Pneumococcal containing vaccines	Included	Included	Included	Included	Included
Shingles (H. Zoster) vaccines		Included	Included	Included	Included
pertussis, inactivated, whole cell vaccines	Included	Included		Included	
DTP (diphtheria, tetanus, and pertussis) combined vaccines		Included	Included	Included	Included
Diptheria, polio tetanus vaccines		Included			
Diphtheria-pertussis-poliomyelitis-tetanus vaccines	Included	Included	Included		
Polio containing vaccines (all types oral/injectable & all valencies)		Included	Included	Included	Included
MMR (measles, mumps and rubella) combined vaccines	Included	Included	Included	Included	Included
MMR (measles, mumps, rubella, varicella) combined vaccines		Included	Included		
MMR (measles, mumps) combined vaccines		Included			
Measles vaccine alone	Included	Included			
Mumps vaccine alone		Included			
Measles, rubella combined vaccines		Included			
Rubella vaccine alone	Included	Included			
Hib (Haemophilus influenzae type b) containing vaccines (alone or combination)		Included	Included	Included	Included
Diphtheria-hepatitis B-pertussis-tetanus vaccines		Included	Included		
Diphtheria-haemophilus influenzae B-pertussis-poliomyelitis-tetanus vaccines	Included	Included	Included		
Haemophilus influenzae B and hepatitis B vaccines		Included			
Hepatitis B vaccines	Included	Included	Included	Included	Included
Diphtheria-haemophilus influenzae B-pertussis-poliomyelitis-tetanus-hepatitis B vaccines	Included	Included	Included		
Diphtheria-haemophilus influenzae B-pertussis-tetanus-hepatitis B vaccines		Included			
Diphtheria-pertussis-poliomyelitis-tetanus-hepatitis B vaccines		Included			
VZV (varicella-zoster virus) vaccines		Included	Included	Included	Included
HPV (human papillomavirus) containing vaccines		Included		Included	Included
Meningitis containing vaccines		Included	Included	Included	Included
Rotavirus containing vaccines		Included	Included	Included	Included
Rabies vaccines		Included			
Typhoid vaccines		Included			

1. There may be underreporting as only vaccines administered in the general practice setting are recorded
2. There may be underreporting for vaccinations given outside of the childhood immunisation programme, since individuals must provide consent for reporting vaccination (with the exception of influenza vaccines)
3. Vaccines administered in travel clinics or pharmacies may not be reported

Supplementary Table S5. Step-by-step variable selection approach in the case-cohort analysis

Data collection and sampling	Identify cases and controls for each DEAP: randomly sample 1,000 sets of matched controls following a repeated random sampling approach.
Univariable testing	- For each of the 1,000 samples, perform logistic regression analysis with the outcome being myocarditis/pericarditis within 30 days following vaccination, and the covariable being each of the variables of interest individually.<br>- Per DEAP, count in how many of the 1,000 samples each variable had confidence interval of the odds ratio that excluded the number 1.<br>- Rank all variables based on the number of confidence intervals that excluded the number 1.
Multivariable variable selection	- For each DEAP and each of the 1,000 samples, start with a model with the clinically established relevant variables (age, sex, and previous SARS-CoV-2 infection)<br>- Add additional variables based on the ranking made in the previous step (i.e., first add the variable with the highest number of confidence intervals of the odds ratio that excluded the number 1).<br>- After each addition, assess model improvement based on the Akaike Information Criterion (AIC) and likelihood ratio between the new and the previous model.<br>- Repeat this process until you have a model that cannot be further improved. This results in 1,000 full models for each DEAP.
Pooling	For the selected model for each of the 1,000 samples per DEAP, estimate the parameters, including odds ratios, for each predictor variable. Use a Rubin's pooling procedure to combine the 1,000 estimates to one overall estimate.
Final model selection	Based on the pooled results, exclude each variable for which the confidence interval of the pooled odds ratio includes 1 from each of the 1,000 models per DEAP. The remaining variables will be kept in the model. Estimate the final model 1,000 times, and pool the estimates, standard errors, odds ratios, and confidence intervals.
